# A Rare Case of Tuberculosis with Sacrococcygeal Involvement Miming a Neoplasm

**DOI:** 10.1155/2016/7286806

**Published:** 2016-07-20

**Authors:** Walid Osman, Meriem Braiki, Zeineb Alaya, Thabet Mouelhi, Nader Nawar, Mohamed Ben Ayeche

**Affiliations:** ^1^Department of Orthopedic Surgery, MES Medical College, Sahloul University Hospital, 4051 Sousse, Tunisia; ^2^MES Medical College, Sahloul University Hospital, 4051 Sousse, Tunisia; ^3^Department of Rheumatology, MES Medical College, University Farhat Hached Hospital, 4051 Sousse, Tunisia; ^4^Department of Orthopedics, MES Medical College, Sahloul University Hospital, 4051 Sousse, Tunisia

## Abstract

Infection of the lumbosacral junction by tuberculosis is quite rare and occurs in only 1 to 2% of all cases of spinal tuberculosis; moreover, isolated sacrococcygeal or coccygeal tuberculosis is much rarer. Failure to identify and treat these areas of involvement at an early stage may lead to serious complications such as vertebral collapse, spinal compression, and spinal deformity. In the present paper, we report an uncommon case of spinal tuberculosis with sacrococcygeal location revealed by a chronic low back pain that was successfully managed. Computed tomography scan and magnetic resonance imaging of the pelvis revealed a lytic lesion affecting both of sacrum and coccyx causing osseous destruction and suggesting a malignant process. A surgical biopsy was performed to establish the tissue diagnosis. Histopathological report confirmed the diagnosis of skeletal tuberculosis. The patient was treated with antibacillary chemotherapy for a period of 9 months. The follow-up period was of 36 months. There was a full recovery and the patient was asymptomatic.

## 1. Introduction

Tuberculosis (TB) has been described as an ancient infectious disease, with evidence being discovered in centuries-old skeletal remains [1, 2].

The proportion of spinal tuberculosis (TB) to all TB cases varied from 1% to 5% [3, 4]. Involvement of the lumbosacral region in spinal tuberculosis is rare, with only few reported cases in the literature [5]. There is only one reported isolated sacrococcygeal lesion [6].

In the present paper, we report an uncommon case of spinal tuberculosis with sacrococcygeal location revealed by a chronic low back pain that was successfully managed.

## 2. Case Presentation

**Figure 1:**
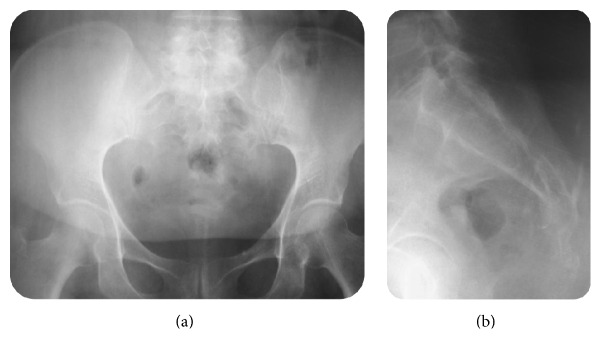
AP and oblique X-rays of pelvis showing fuzziness of the sacrococcygeal region with irregular margins.

**Figure 2:**
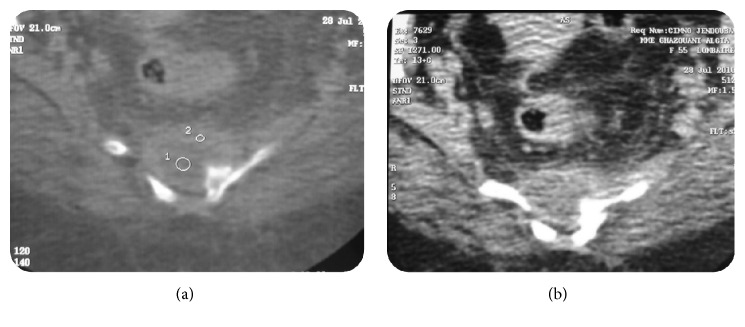
Contrast-enhanced computed tomography (CT) scans of the patient revealing presacrococcygeal lytic process.

**Figure 3:**
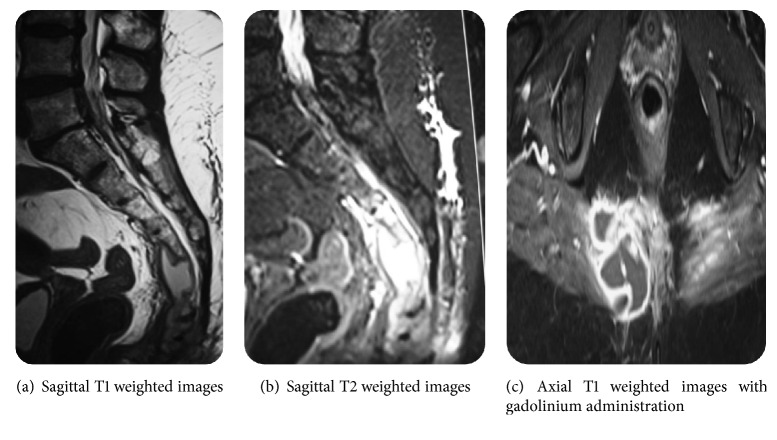
Magnetic resonance images of the patient. Revealing a process of hypointense signals in T1W images (a) and hyperintense signals in T2W images (b), the lesion was moderately enhanced in its periphery after gadolinium administration (c).

A 55-year-old woman without medical or surgical history of interest was referred to our team with 1-year history of isolated lower back pain. She reported neither fever nor recent weight loss; there was no tuberculosis contagion. On physical examination, the patient was afebrile. Palpation of the sacrococcygeal region was too painful. Respiratory, cardiovascular, and abdominal system examination was normal. Higher mental functions and cranial nerve examination were normal. There were no meningeal signs. Motor and sensory examination in the lower limbs was normal. There was no bowel or bladder incontinence. Biologic parameters were within normal limits. Chest X-ray was normal. These were followed up by AP and oblique X-ray of pelvis (Figure 1) which were not suggestive of any lesion and showed blurry margins of the sacrococcygeal region. Initial CT scan of the pelvis (Figure 2) revealed a large hypodense heterogeneous solid lesion with enhanced peripheral portions after contrast administration, affecting both of sacrum and coccyx causing osseous destruction and suggesting a malignant process. The magnetic resonance imaging (MRI) (Figure 3) was performed showing a process of hypointense signals in T1W images and hyperintense signals in T2W images. The lesion was moderately enhanced in its periphery after gadolinium administration. The spread of lesion to right piriformis muscle and large gluteal muscles was noted.

Based on these radiological findings, a malignant process such as chordoma was suspected.

**Figure 4:**
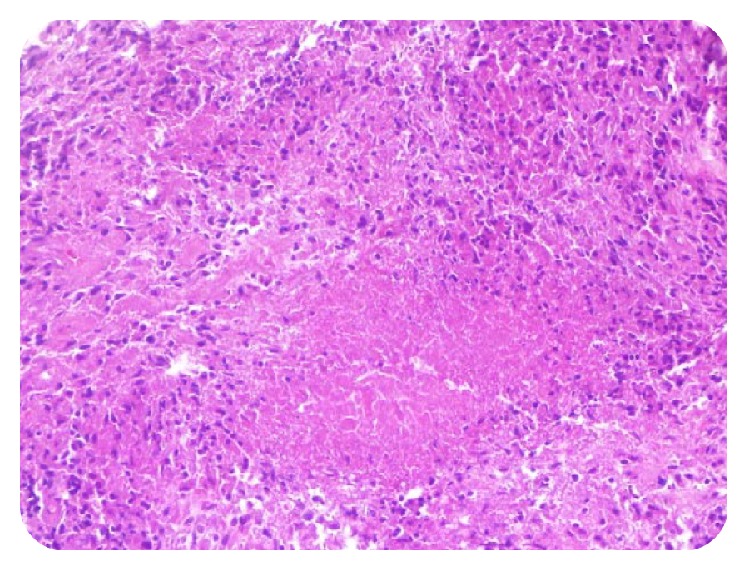
Histological examination revealing caseating granulomatous inflammation.

**Figure 5:**
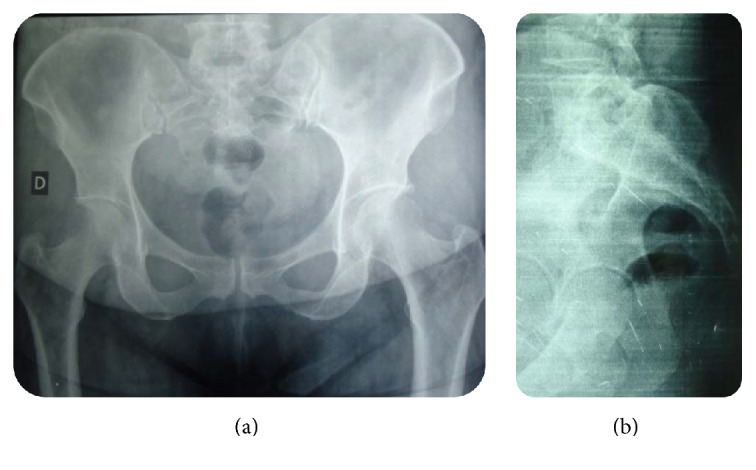
Follow-up AP and oblique X-rays of pelvis at 3 years showing no evidence of recurrence.

The patient underwent a surgical intervention and an open biopsy was performed to establish the tissue diagnosis. Histopathological report (Figure 4) confirmed the diagnosis of skeletal tuberculosis by showing tuberculous granuloma with central caseous necrosis surrounded by epithelioid cells and Langhans type giant cells. The patient was treated with antibacillary chemotherapy for a period of 9 months. Treatment was initiated with a four-drug antibiotic regimen (isoniazid, rifampicin, pyrazinamide, and ethambutol) for initial intensive phase of two months followed by continuation phase with two-drug regimen (isoniazid and rifampicin) for next 7 months with a favorable evolution. The follow-up period was of 36 months. There was a full recovery and the patient was asymptomatic at the last follow-up after 3 years with standard radiographs showing no evidence of recurrence (Figure 5).

## 3. Discussion

Tuberculosis still remains one of the most pressing health problems in the developing world, and tuberculosis of the spine occurs by hematogenous spread of infection from a pulmonary or extrapulmonary site; pulmonary infection is detected in around 50% of cases of spinal tuberculosis. More rarely, the condition may be encountered in the absence of a pulmonary infection [7].

The infection begins in subchondral bone and spreads slowly to the intervertebral disk space and the adjacent vertebral bodies, commonly in the lower dorsal and upper lumbar spine [8].

Failure to identify and treat these areas of involvement at an early stage may lead to serious complications such as vertebral collapse, spinal compression, and spinal deformity [8, 9].

Infection of the lumbosacral junction by tuberculosis is quite rare and occurs in only 1 to 2% of all cases of spinal tuberculosis; moreover, isolated sacrococcygeal or coccygeal tuberculosis is much rarer [7].

The sacrum is an uncommon site for tuberculosis involving the spine. In a review of 107 patients of tuberculous spondylitis by Lifeso et al. [10], no patient had lumbosacral and sacrococcygeal involvement. Dayras et al. [11] reported first case of isolated sacral tuberculosis with lower back pain. In 2004, Wellons et al. [12] presented a case of sacral tuberculosis with lower back pain and difficulty in walking with bilateral involvement of lower limbs.

However, the reasons for the low incidence of lumbosacral or sacrococcygeal tuberculosis have not been exactly elucidated [7].

The single reported case of sacrococcygeal tuberculosis presented as an anal fistula [6]. Our patient with isolated sacrococcygeal involvement is probably the second described in the literature.

A confident diagnosis of skeletal involvement in tuberculosis is often difficult as the clinical presentation is nonspecific. Constitutional symptoms and nonspecific back pain are the predominant complaints. Thus, a delayed diagnosis is common [13]. Isolated sacral tuberculosis usually presents as chronic back pain in adults and discharging sinuses or abscess formation in children, with or without neurological deficit [7].

Briefly, in the literature, it was reported that isolated sacral or coccygeal tuberculosis is generally revealed by back pain without neurological deficit. That was compatible with the present case in which the patient was complaining of isolated chronic low back pain.

Neurological deficit is relatively uncommon in isolated sacral tuberculosis [14]. Because the sacral nerve roots are protected by bone, the incidence of neurological symptoms is relatively low [7].

Imaging findings in musculoskeletal TB are often nonspecific: in fact, plain radiographs are extremely insensitive and do not detect vertebral involvement until at least 50% of a vertebra is destroyed [7]. The first sign may be demineralization of the endplates with resorption and loss of dense margins [13]. Pre- and parasacral collection with destruction of the sacrum is seen on CT scan. MRI is the most sensitive modality for early diagnosis and complete delineation of the disease [8]. It usually reveals diffuse marrow edema which is hypointense on T1 and hyperintense on T2 weighted images; gadolinium contrast-enhanced MRI shows the enhancement of granulation tissue [14]. However, the MRI appearance of infection caused by* M. tuberculosis* is similar to the appearance of spinal neoplasms [15]. Consequently, bone imaging can notably be prone to miss this disease: pyogenic osteomyelitis and neoplasm such as chordoma or osteogenic sarcoma were included in the differential diagnosis [7].

As tuberculosis lesions may be mistaken for other infectious diseases or neoplasms, fresh tissue should be obtained for culture and biopsy [7].

The diagnosis of tuberculosis is confirmed by the histopathological study [16]. We presented a case of a neurologically intact patient with spinal tuberculosis because of the uncommon anatomical location of her lesion as well as radiological features suggesting a neoplasm such as chordoma. Surgical biopsy was indicated. Pathologic examination of biopsy specimens confirmed the diagnosis.

Regarding treatment of spinal tuberculosis, various chemotherapy protocols have been proposed. Medical treatment involves a combination of four drugs: rifampicin, isoniazid, pyrazinamide, and ethambutol for 2 months followed by bitherapy [3]. Thus, many workers prescribe chemotherapy for 6 months, while some continue it for 9 to 12 months [16, 17]. Excellent response of vertebral tuberculosis to multidrug therapy prevents the need for surgical management [16]. Our patient had a complete recovery with antitubercular treatment and bed rest.

Surgery may be necessary because of signs of neurological compression during extensive destruction of several vertebral bodies with spinal deformity or to evacuate an abscess that is resistant to medical treatment.

The prognosis of sacral tuberculosis is good, if a rapid and correct diagnosis is made and adequate treatment is provided [17].

This pathology should always be suspected in any process of the lytic sacrum or coccyx, especially in endemic areas of tuberculosis, to prevent or at least reduce the morbidity of this disease, which is generally curable [16].

## 4. Conclusion

Isolated sacrococcygeal tuberculosis is exceptional, and it should be kept in mind in isolated low back pain associated with any process of the lytic sacrum and coccyx. It is necessary to make a rapid and correct diagnosis in order to provide adequate management for this disease which has an excellent response to antitubercular treatment.
